# Family caregiver constructs and outcome measures in neuro-oncology: A systematic review

**DOI:** 10.1093/nop/npac058

**Published:** 2022-07-20

**Authors:** Florien Boele, Caroline Hertler, Linda Dirven, Karin Piil, Paula Sherwood

**Affiliations:** Leeds Institute of Medical Research at St James’s, St James’s University Hospital, University of Leeds, Leeds, UK; Faculty of Medicine and Health, Leeds Institute of Health Sciences, University of Leeds, Leeds, UK; Department of Radiation Oncology, Competence Center Palliative Care, University Hospital Zurich and University of Zurich, Zurich, Switzerland; Department of Neurology, Leiden University Medical Center, Leiden, The Netherlands; Department of Neurology, Haaglanden Medical Center, The Hague, The Netherlands; Department of Oncology, Centre for Cancer and Organ Diseases, Copenhagen University Hospital, Rigshospitalet, Copenhagen, Denmark; Department of Public Health, Aarhus University, Aarhus, Denmark; School of Nursing, University of Pittsburgh, Pittsburgh, Pennsylvania, USA

**Keywords:** burden, distress, family caregiver, outcome measures, quality of life, unmet needs

## Abstract

**Background:**

As a first step to reach consensus on the key constructs and outcomes in neuro-oncology caregiver research, we performed a systematic review to evaluate the constructs that are being evaluated in research studies and how these have been assessed.

**Methods:**

All peer-reviewed publications with primary data reporting on outcomes of family caregivers of adult primary brain tumor patients were eligible. Electronic databases PubMed/Medline, Embase, Web of Science, Emcare, Cochrane Library, and PsycINFO were searched up to September 2021. Using Covidence, title and abstract screening, full-text review, and data extraction were done by two researchers independently, with a third guiding consensus. Constructs as reported in each study, and how these were assessed were the primary result.

**Results:**

Searches yielded 1090 unique records, with 213 remaining after title/abstract screening. Of these, 157 publications met inclusion criteria, comprising 120 unique studies. These originated from 18 countries and were published between 1996 and 2022. Most were observational (75%) cross-sectional (61%) studies, reporting on quantitative methods (62%). Twenty-seven different constructs were assessed and mapped along the Caregiver Health Model (CGHM) categories, namely, caregiver health, needs, tasks, beliefs and attitudes, and environment. Seventeen questionnaires were used >2 times to measure the same construct, with the vast majority of questionnaires only used across one or two studies.

**Conclusions:**

Neuro-oncology caregiving research is a field gaining traction, but lags behind in clear definition of key constructs, and consistency in assessment of these constructs. Developing consensus or guidance will improve comparability of studies, meta-analyses, and advance the science more quickly.

Patients with primary brain tumors have a high symptom burden which combines the most difficult aspects of cancer (eg, sudden diagnosis and aggressive treatment) and neurological conditions such as dementia (eg, neuropsychiatric and behavioral changes).^1^ Patients commonly rely on family caregivers for practical and emotional support, which can have negative mental and physical consequences for caregivers.^2^ Maintaining optimal caregiver well-being is critical not only to allow caregivers to support patients throughout the disease trajectory, but also to prevent caregivers needing to seek professional care themselves as a result of chronic stress, physical strain, and poor self-care.^3^

Even if support needs are identified, caregiver support that meets specific neuro-oncology needs is difficult to access. Neuro-oncology caregivers consistently report barriers to obtaining acceptable, suitable, and effective support, including finding it difficult to ask for help, wanting to actively request support but not knowing how to access support, and support often not being neuro-oncology specific.^4,5^ This is echoed by health professionals, who identified system-level barriers including limited resources, funds, and availability of staff with brain-tumor specific training.^6^

Several recent systematic reviews have collated the current evidence-base for supportive interventions for neuro-oncology caregivers—including those who are bereaved.^7–9^ A Cochrane systematic review from Boele et al. identified eight small-scale trials (range *N* = 13–56). The interventions tested varied in content and delivery, with some aimed at improving caregiver well-being directly,^10–13^ and some indirectly through a dyadic patient-caregiver support intervention.^14–17^ Some evidence for improvements in caregiver distress, feelings of mastery, and quality of life were found yet the overall quality of evidence was low, making reliable conclusions on intervention effectiveness difficult.^7^ A more recent systematic review by Heinsch et al. included additional evidence from nonrandomized trials. The 13 intervention studies included were similarly diverse in content and delivery and showed some effect in reducing depressive symptoms and improving mastery in caregivers.^8^ Both reviews call for fully powered randomized controlled trials to enhance the quality of the evidence. In addition, both reviews indicate that there is a distinct lack of consensus on which constructs are key to measure in neuro-oncology caregivers, and which instruments are the most appropriate to assess these constructs. These issues preclude study comparisons and ultimately, meta-analyses, which are considered to provide the highest level of evidence for implementing changes to practice. Moreover, it generates research waste and slows research progress overall.^18^

As a first step to reach consensus on the key constructs and outcome measures in neuro-oncology caregiver research, we have undertaken a comprehensive systematic review of studies with neuro-oncology caregivers of adult primary brain tumor patients. We focus on the constructs being evaluated, and the outcome measures used to evaluate these constructs. This work will ultimately highlight which constructs have been well-researched and are linked with validated outcome measures, and which domains are currently not covered by existing literature or outcome measures. Identifying these gaps is vital to guide instrument development and implementation for neuro-oncology caregiver research, and ultimately clinical practice. This work lays the groundwork for future efforts to recommend a core outcomes set for use in neuro-oncology caregiver studies.

## Materials and Methods

### Study Design

This systematic literature review aimed at evaluating what constructs and outcome measures have been used in peer-reviewed studies focusing on family caregivers of adult patients with primary brain tumors. Here, “constructs” were defined as the subject matter that studies aim to measure; “outcome measures” were defined as the instrument (or, in case of qualitative research, method) used to assess a construct. Where applicable, we followed PRISMA guidelines.^19^

### Search Strategy

An extensive literature search for articles published up to September 20, 2021 (there was no specific start date) was conducted in the following electronic databases: PubMed/Medline, Embase, Web of Science, Emcare, Cochrane Library, and PsycINFO. The searches consisted of a combination of terms for (1) informal caregivers of adult patients diagnosed with primary brain tumors, (2) outcomes/constructs, and (3) outcome measures. Development of search terms was guided by terminology used in previous research, existing frameworks, and expert opinion. Supplementary Material 1 includes the search string for PubMed, which was adapted for the other databases.

### Study Selection

All citations identified in the searches were imported into Covidence,^20^ after removal of any duplicate publications. Articles were included according to the criteria displayed in Table 1. Citations were assessed in two stages. First, titles and abstracts were screened against inclusion criteria by two reviewers (F.B. and P.S.) independently. Then, full texts of potentially relevant articles were added into Covidence and assessed by two reviewers (F.B., P.S., L.D., K.P., and/or C.H.) independently. At both stages, differing opinions were discussed until agreement was reached, if needed guided by a third researcher. All decisions were coded and recorded in Covidence. Reviewers were not blinded to journal titles, authors or institutions.

**Table 1:** Inclusion and exclusion criteria for study selection

Inclusion criteria	Exclusion criteria
*Types of participants*	
Studies covering adult caregivers (current or bereaved) of adult patients with primary brain tumors	Studies involving caregivers under <18 years of age
In samples which included caregivers of both young and adult patients, studies were included if adult (18+) patients made up at least 90% of the sample	Studies involving only caregivers of childhood brain tumor patients or childhood brain tumor patients who are now adults
Studies reporting on mixed caregiver populations (eg, acquired brain injury or cancer) were included, as long as primary brain tumors were part of the sample	Studies involving only caregivers of patients with metastatic brain tumors
*Types of studies*	
Peer-reviewed studies of any design, reporting on caregiver outcomes and/or outcome measures	Reviews (not original research)
	Non-peer reviewed studies
	Conference abstracts
	Grey literature
	Studies in which caregivers are included as the proxy reporter for patients
*Geographical coverage*	
Any setting, any country	N/A
*Language*	
Published in English, Dutch, Danish, French, or German	Published in languages other than English, Dutch, Danish, French, or German

### Data Extraction

A data extraction form was drafted and piloted in Covidence prior to data extraction. Data extracted included basic study information (title, author, year, country where data were collected, and funding), study characteristics (eg, study design and aims), constructs and how these were assessed, and study population details (caregiver participants, patient populations, and sociodemographic information). Missing data were recorded as “not known”; study authors were not contacted. The actual study results were not extracted as this review is focused on identifying constructs and outcome measures only. Therefore, we also did not complete a quality assessment of included studies. All authors contributed to data extraction, with details on each study extracted by two individual reviewers (F.B., P.S., L.D., K.P., and/or C.H.). A third author then performed a consensus check for each publication before data extraction was finalized.

### Synthesis

Extracted data were processed in SPSS version 26. Based on funding source, author, and country, publications were linked to unique studies where appropriate. Characteristics of included studies were analyzed with descriptive statistics. Nonparametric tests were done to explore associations between year of publication and key study characteristics (aspects of study design and whether caregiver outcomes were a primary or secondary aim of the study), with *P* < .05 indicating statistical significance. To synthesize the review findings, we first listed all constructs assessed in included studies. These were then grouped for overlap or relatedness to other constructs along the Caregiver Health Model,^21^ originally developed for family caregivers of elders. This model places caregiver health at the center, impacted by caregiver needs, tasks, beliefs and attitudes, and health promotion behaviors (eg, nutrition, physical activity, and other lifestyle choices) as determinants, and considers this within the environmental context. We describe how constructs were assessed (eg, using qualitative methods or quantitatively using a questionnaire or instrument, and if so, which). We report how constructs were assessed exactly as described in the publications, and do not make a judgment of the suitability thereof (eg, if a study described assessing quality of life using a distress scale, this is what we report even though it may not cover all aspects of the multidimensional quality of life construct). For each identified construct, we tabulate the more common ways of assessment (ie, questionnaires or instruments used in more than two studies).

## Results

**Fig. 1:**
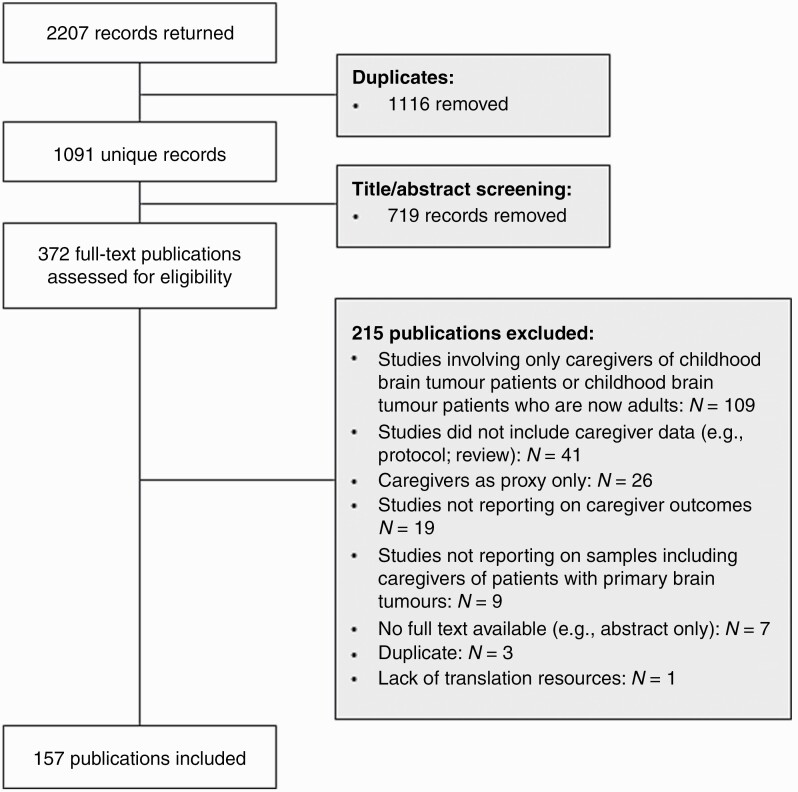
Flow diagram of study selection.

Following deduplication, the literature search produced 1091 unique records. Through title and abstract screening, 719 records were removed, leaving 372 for full-text review. Of these, 157 met the inclusion criteria as outlined in Table 1 (see Supplementary Material 2 for a full list of references). The flow diagram of study selection is shown in Figure 1.

### Characteristics of Included Publications

Table 2 displays characteristics of publications included in the review. These were published in peer-reviewed journals between 1996 and 2022, with the majority (*N* = 82, 52%) published after 2015. These 157 publications correspond to 120 unique studies. Study data were collected in 18 different countries across the continents of Europe (*N* = 51, 43%), North America (*N* = 44, 37%), Oceania (*N* = 15, 13%), and Asia (*N* = 10, 8%). Most were observational studies (*N* = 90, 75%) and used quantitative research methods (*N* = 74, 62%) and a cross-sectional design (*N* = 73, 61%). For 88 studies (73%), caregiver outcomes were part of the primary study aims either on their own or as part of the patient-caregiver dyad. More recent studies more frequently included caregiver outcomes as part of the primary (median year = 2015) rather than secondary study aim (median year = 2013, *P* = .03), and more often used quantitative (median year = 2016) or mixed methodology (median year = 2017) rather than qualitative methodology (median year = 2012, *P* = .01).

**Table 2:** Study and publication characteristics

Study characteristics		Unique studies *N* = 120	Publications *N* = 157
Study focus	Caregiver outcomes in primary aims	88 (73.3%)	119 (75.8%)
	Caregiver outcomes in secondary aims	32 (26.7%)	38 (24.4%)
Year of publication (median, range)		2015, 1996–2022	2015, 1996–2022
Country of data collection	United States	38 (31.7%)	56 (35.7%)^a^
	Australia	15 (12.5%)	23 (14.6%)
	Germany	14 (11.7%)	15 (9.6%)
	Sweden	8 (6.7%)	12 (7.6%)
	Denmark	7 (5.8%)	10 (6.4%)
	United Kingdom	6 (5.0%)	7 (4.5%)
	Canada	6 (5.0%)	6 (3.8%)
	The Netherlands	4 (3.3%)	5 (3.2%)
	France	3 (2.5%)	4 (2.5%)
	Italy	4 (3.3%)	4 (2.5%)
	India	3 (2.5%)	3 (1.9%)
	Singapore	2 (1.7%)	2 (1.3%)
	Turkey	2 (1.7%)	2 (1.3%)
	Austria	2 (1.7%)	2 (1.3%)
	Belgium	2 (1.7%)	2 (1.3%)
	China	2 (1.7%)	2 (1.3%)
	Switzerland	1 (0.9%)	1 (0.7%)
	South Korea	1 (0.9%)	1 (0.7%)
Study design	Experimental study: randomized controlled trial	6 (5.0%)	9 (5.7%)
	Experimental study: nonrandomized trial with control group	2 (1.7%)	2 (1.3%)
	Experimental study: intervention study without control group	16 (13.3%)	17 (10.8%)
	Observational study: cohort	90 (75.0%)	123 (78.3%)
	Observational study: case control	6 (5.0%)	6 (3.8%)
Data collection	Cross-sectional	73 (60.8%)	90 (57.3%)
	Longitudinal	47 (39.2%)	67 (42.7%)
Type of information collected	Quantitative	74 (61.7%)	94 (59.9%)
	Qualitative	29 (24.2%)	41 (26.1%)
	Mixed methods	16 (13.3%)	21 (13.3%)
	Unclear	1 (0.9%)	1 (0.7%)
Sample size	Full caregiver sample	M = 80.9, SD = 164.4, range 1-1580^b^	M = 75.7, SD = 145.3, range 1-1580^b^
	In mixed samples, proportion of neuro-oncology caregivers	M = 25.4%, SD = 25.4%, range 1%–78%^c^	M = 24.4%, SD = 25.1%, range 1%–78%^d^
Study population	Caregivers only	39 (32.5%)	55 (35.0%)
	Patients and caregivers	78 (65.0%)	99 (63.1%)
	Caregivers, professionals, and patients	3 (2.5%)	3 (1.9%)
Disease group	Primary malignant brain tumors	50 (41.7%)	71 (45.2%)
	Primary brain tumors	39 (32.5%)	52 (33.1%)
	Brain tumors (not specified, or mixed with other intracranial lesions)	8 (6.7%)	9 (5.7%)
	Mixed neurological or oncological populations	23 (19.2%)	25 (15.9%)
Patient age group	Adults only	108 (90.0%)	143 (91.1%)
	Mixed pediatric/adult patients where adults are >90% of sample	2 (1.7%)	2 (1.3%)
	Unclear	9 (7.5%)	12 (7.6%)
Caregiver age	Reported (full sample)	92 (76.7%)	122 (77.7%)
	Reported (neuro-oncology specific)	76 (63.3%)	103 (65.6%)
Caregiver gender	Reported (full sample)	98 (81.7%)	127 (80.9%)
	Reported (neuro-oncology specific)	84 (70.0%)	117 (72.0%)
Caregiver-patient relationship	Reported (full sample)	96 (80.0%)	124 (79.0%)
	Reported (neuro-oncology specific)	79 (65.8%)	110 (70.1%)

^a^One publication presented data collected in two countries, both U.S. and the Netherlands—this was only counted under U.S.

^b^Data not reported in one study/publication.

^c^Data not reported in seven studies.

^d^Data not reported in nine publications.

Across the 120 studies, overall caregiver sample sizes ranged from 1 to 1580 (mean = 80.9, SD = 164.4). In studies with mixed samples (eg, oncology or neurology), on average 25% (range 1%–78%) of the sample consisted of neuro-oncology caregivers. Caregiver age for overall caregiver samples was reported in 92 studies (77%), either as a range, mean, median, or in categories. Due to heterogeneity of reporting, no meaningful information about the caregiver age can be provided. Within overall samples, on average 66% of caregivers were women (range 30%–100%) and 72% were spouses (range 24%–100%). Within neuro-oncology–specific caregiver samples, on average 66% of caregivers were women (range 40%–100%), and 73% were spouses (range 29%–100%).

### Constructs and Outcomes

**Fig. 2:**
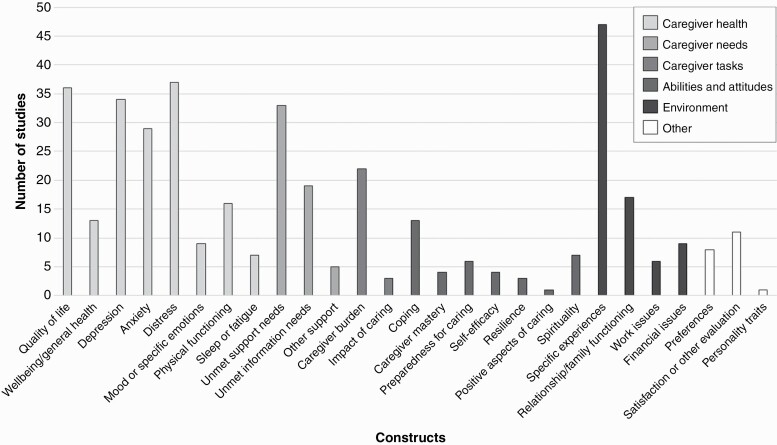
Frequency of constructs covered in studies exactly as described in the publications, grouped along Caregiver Health Model (CGHM) categories.

Across included publications, 27 constructs were assessed in caregivers (see Figure 2). These were grouped into the following categories: (1) caregiver health; (2) caregiver needs; (3) caregiver tasks; (4) caregiver beliefs and attitudes; and (5) environment or other. No constructs were found to fit under the final domain of the CGHM (caregiver health promotion behaviors). For each category we described which instruments were used to assess the constructs of interest. Per construct we list the more frequently used (in >2 studies) questionnaires or tools (see Table 3). Supplementary Material 3 includes a full list of questionnaires or tools.

**Table 3:** Overview of the instruments used in >2 studies to assess constructs in the neuro-oncology caregiving literature

1. Caregiver health			
Construct	Assessed with…	Reported in *N* studies	Designed for…
Quality of Life	Caregiver Quality of Life Index-Cancer Scale (CQOLC)	12	Quality of life of caregivers (cancer caregiver specific)
	MOS Short Form 12/36 (SF-12; SF-36)	10	Health-related quality of life (generic)
	European Organisation for Research and Treatment of Cancer Quality of Life Questionnaire Core-30 (EORTC QLQ C30)	3	Health-related quality of life (cancer patient specific)
Depression	Hospital Anxiety and Depression Scale (HADS)	16	Symptoms of anxiety and depression in medically ill patients
	Center for Epidemiological Studies-Depression Scale (CES-D; shortened or full length)	9	Depressive symptoms (general population)
Anxiety	HADS	15	Symptoms of anxiety and depression in medically ill patients
	State-Trait Anxiety Inventory (STAI)	3	State and trait anxiety
Psychological distress	Distress Thermometer (DT)	12	Screen for distress in cancer patients
	General Health Questionnaire (GHQ-12)	4	Identifying nonpsychotic and minor psychiatric disorders
	Perceived Stress Scale (PSS)	3	Perception of stress (general population)
Physical functioning	MOS Short Form 12/36 (SF-12; SF-36)	4	Health-related quality of life (generic)
Sleep or fatigue	Pittsburgh Sleep Quality Index (PSQI)	4	Sleep quality and disturbances (generic)
**2. Caregiver needs**			
**Construct**	**Assessed with…**	**Reported in *N* studies**	**Designed for…**
Unmet support needs	Partner and Caregiver Supportive Care Needs Scale (SCNS-P&C)	3	Supportive care needs (cancer caregiver specific)
**3. Caregiving tasks**			
**Construct**	**Assessed with…**	**Reported in *N* studies**	**Designed for…**
Burden	Zarit Burden Interview (ZBI)	5	Caregiver burden (dementia)
	Caregiver Reaction Assessment (CRA)	3	Reactions of family members (elderly persons with physical impairments; dementia; cancer)
**4. Caregiver beliefs and attitudes**			
**Construct**	**Assessed with…**	**Reported in *N* studies**	**Designed for…**
Mastery	Caregiver Mastery Scale (based on Pearlin and Schooler)	3	Caregiver mastery (generic)
Preparedness	Preparedness for Caregiving Scale	5	How well-prepared respondents believe they are in caregiving (generic)

#### Caregiver health

Constructs related to caregiver health were defined in publications as quality of life, well-being or general health, emotional and physical health, and sleep or fatigue.

Overall quality of life was assessed in 36 studies, using 15 different questionnaires. The most frequently used instruments are the Caregiver Quality of Life Index-Cancer (CQOLC) (*N* = 12), and either the 12- or 36-item versions of the MOS Short Form (SF-12 or SF-36; *N* = 10). Three publications reported using the European Organisation for Research and Treatment of Cancer Quality of Life Core-30 questionnaire (EORTC QLQ-C30). Three studies assessed quality of life qualitatively. Well-being or general health was assessed in 13 studies using nine different questionnaires, with three studies using qualitative methods. Questionnaires used typically overlap for those used to assess quality of life (eg, SF-36 and CQOLC), but study-specific tools were also used (*N* = 3).

Caregiver emotional health was covered in studies as either depressive symptoms, anxiety, distress, mood, or specific emotions such as grief, anger, and death anxiety. Depression as a construct was assessed in 34 studies, using 10 different questionnaires. Most frequently, the Hospital Anxiety and Depression Scale (HADS; *N* = 16) or Center for Epidemiological Studies-Depression Scale (CES-D; *N* = 9) were used. Anxiety was assessed in 29 studies, using 12 different questionnaires (most frequently the HADS [*N* = 15]). Psychological distress was assessed in 37 studies using 19 different questionnaires. Most frequently the Distress Thermometer (*N* = 12) was used, but the General Health Questionnaire (GHQ-12; *N* = 4) and the Perceived Stress Scale (PSS; *N* = 3) were also reported to be used to assess distress. Furthermore, nine studies reported on mood or specific emotions such as anger, grief, or death anxiety, four using different quantitative instruments with five other studies describing the construct qualitatively.

Caregiver physical health was covered in 16 studies as physical functioning, with seven studies reporting on sleep or fatigue. Physical functioning was assessed using eight different questionnaires, with the SF-12 or SF-36 most commonly used (*N* = 4). Qualitative methods (*N* = 3) and objective measures (*N* = 5) such as through blood markers or cortisol levels were also reported on. Sleep or fatigue was measured with seven questionnaires, with the Pittsburgh Sleep Quality Index (PSQI) most commonly used (*N* = 4). Objective measures using a wrist motion sensor were reported on in two studies.

#### Caregiver needs

Caregiver needs were described in publications as either (unmet) support needs, information needs, or other needs including social support needs.

Unmet support needs were assessed in 33 studies, the most frequently using qualitative methods only (*N* = 14). Furthermore, 20 different questionnaires were reported on, with none used in more than two studies. Information needs were assessed in 19 studies, again the most frequently using qualitative methods (*N* = 10). Nine different questionnaires were also used, all of these only once. “Other support,” reflecting, for example, social support needs, was assessed in five studies, using two different questionnaires, none used more than once, and qualitatively in three studies.

#### Caregiver tasks

Across studies, caregiver tasks were assessed as either caregiver burden (*N* = 22) or impact of caring (*N* = 3). Twelve different questionnaires were used. The Zarit Burden Interview was used in five studies, and the Caregiver Reaction Assessment in three studies; other questionnaires were only used once or twice.

#### Caregiver beliefs and attitudes

Constructs falling under caregiver beliefs and attitudes included coping, caregiver mastery, preparedness for caring, self-efficacy, resilience, positive aspects of caring, and spirituality.

- *Coping* (*N* = 13) was assessed with six different questionnaires, none used more than twice.
- *Caregiver mastery* (*N* = 4) was assessed with the Caregiver Mastery Scale based on Pearlin and Schooler (*N* = 3).
- *Preparedness for caring* (*N* = 6) was the most often assessed with the Preparedness for Caregiving Scale (*N* = 5).
- *Self-efficacy* (*N* = 4) was assessed with three different questionnaires, none used more than twice.
- *Resilience* (*N* = 3) was measured with three different instruments, all only once.
- *Positive aspects of caring* (*N* = 1) was assessed with a quantitative instrument in one study.
- *Spirituality* (*N* = 7) was the most often assessed qualitatively (*N* = 4), while three studies used a quantitative instrument, both only once.

#### Environment

In the CGHM, environment provides the context within which to evaluate caregiver health. We identified 47 studies that assessed specific experiences not already covered within the categories described above. These experiences were for example, reflections on patient treatment pathways, end-of-life care, and decision-making. Most often this was assessed using qualitative methods (*N* = 33). A further 12 questionnaires were also used, of which four were study-specific—all only used once or twice. In addition, relationship or family functioning was assessed in 17 studies. Eleven different questionnaires were reported on, none used more than twice. Issues around paid work were assessed in six studies, and financial issues were assessed in nine studies. Most commonly, study-specific questionnaires were used, with no quantitative instrument used more than once.

#### Other constructs

Caregiver preferences, for example, type of support or communication preferences, were assessed in eight studies with four using qualitative methods and four a study-specific questionnaire. Furthermore, 11 studies covered caregivers’ satisfaction or evaluation scores of some kind, for example, related to a supportive intervention. This was commonly done using qualitative methods (*N* = 3). Finally, one study assessed caregiver personality traits, specifically neuroticism (*N* = 1) using a quantitative instrument.

## Discussion

This systematic review analyzed all studies of family caregivers of patient samples which included adult patients with primary brain tumors, up to September 2021. Across 120 studies, 157 publications covered 27 different constructs. In addition, over 45 studies covered specific caregiver experiences, preferences, or satisfaction or evaluation of care or interventions. Qualitative methods are quite common in neuro-oncology caregiver publications, although more recent publications were apt to use quantitative or mixed methods designs. This indicates that the field of neuro-oncology caregiving research is moving from purely describing constructs toward quantifying them. Yet, a wide range of quantitative instruments is reported with a substantial proportion of instruments only used in one or two studies. Just seventeen unique questionnaires were used across more than two studies to assess the same construct(s). Although the present systematic review bears resemblance to a previous effort which identified 215 patient-reported outcome measures (the majority only used once or twice) across 571 published and 194 unpublished studies to assess neuro-oncology patient functioning and well-being,^22^ the difference in volume of research is striking and emphasizes that caregiver research may not be considered an equal priority. More consistent use of validated tools will increase the possibility of comparing studies and will facilitate meta-analyses, helping reach higher-level evidence.

The present review does not evaluate whether these instruments were designed or validated for use in neuro-oncology caregiver populations. For example, while commonly used in neuro-oncology caregivers, the HADS was developed for use in medically ill populations. Whether this questionnaire has good psychometric properties for use in neuro-oncology caregiver populations specifically, for example, in terms of content validity, reliability, or responsiveness, would need to be investigated. Similarly there could be questionnaires that have been used less frequently, but for which there is evidence of adequate psychometric properties in the neuro-oncology caregiver population—for example, the Locke-Wallace Short Marital Adjustment Test,^23^ and the Caregiver Needs Screen.^24^ Therefore, future efforts should be directed toward evaluating the suitability of the identified outcome measures for neuro-oncology caregiving research. One conceptual difficulty to overcome prior to starting this endeavor is that while some constructs appear well-established (eg, burden and quality of life), these are not always well-defined. For example, some burden instruments may assess aspects of burden that are not covered by other instruments designed to assess the same construct. Overlap in aspects of separate constructs is also possible, with, for example, psychological distress having likely overlap with both anxiety and depression, as well as emotional domains of quality of life. For each construct frequently covered in neuro-oncology caregiving research, obtaining consensus on what this construct should cover would enhance the clarity of evidence.

The CGHM was used to map constructs and outcomes found in included studies.^21^ While not developed for neuro-oncology caregiving, the model approaches caregivers comprehensively, placing their health at the center impacted by needs, tasks, beliefs and attitudes, and health promotion behaviors while taking environmental factors into account. There is a noticeable gap in the neuro-oncology caregiver literature related to health promotion behaviors. To a limited extent, these may have been captured in the broad “experiences” construct, as studies which evaluated, for example, intervention use will have been grouped here. There may also be some overlap with the construct of coping. The emphasis on more positive, empowering constructs including health promotion behaviors reflects trends in medical, nursing, and psychology research, and funding priorities. In recent years, more negative, sometimes medicalizing constructs such as depression or burden are viewed less favorably. This development is interrelated with the growing popularity of eHealth and self-management interventions,^25^ which often give a greater level of control with the participant while focusing less on mitigating symptom burden. With this, health promotion behavior outcomes will likely become more established. It is therefore likely that constructs and outcomes related to health promotion behaviors will grow in popularity.

Due to the scale of the current systematic review, we made particular effort to minimize bias by involving two authors in both title/abstract and full-text screening, with discrepancies resolved through a third author if needed. Similarly, we had two authors extracting data from all 157 publications, with a third author performing a consensus check. The study team includes authors from five different countries and the research was done without financial support, limiting bias. Study selection criteria were kept purposefully broad, making this a robust but also comprehensive piece of work. Yet despite its strengths, this systematic review also has its limitations. Notably, there remains the possibility of errors in grouping publications into studies based on author name, country, and funding. Furthermore, while aiming to be highly inclusive in study selection, we employed language restrictions for feasibility purposes. The impact of the language restrictions is likely very limited as we only excluded one full-text paper on this basis, which was published in Chinese.^26^ Finally, new evidence is constantly emerging so it should be noted that this systematic review only includes peer-reviewed articles published (online, possibly before copy-editing) by September 20, 2021. Therefore, findings should be interpreted with some caution.

We highlight gaps for further research based on our review findings, inviting interested readers to contact us for research collaborations and to join the International Neuro-oncology Caregiver Consortium (INCC). First, while some constructs appear well-established (eg, burden, quality of life), these are not always well-defined. For each construct frequently covered in neuro-oncology caregiving research, obtaining consensus on what this construct should cover would enhance the clarity of evidence. Second, some constructs conceptually overlap. For example, caregiver mastery and preparedness for caregiving, or coping and self-efficacy or resilience, may be highly correlated. Obtaining a clear overview of overlap between constructs, would therefore be beneficial to the field. Third, the field would benefit from evaluation of psychometric properties of existing instruments matched to constructs, or where these are not available, development of new instruments suitable for use in neuro-oncology caregiver populations. Fourth, reporting of basic sociodemographic information (age, gender, and relationship to patient) was incomplete in roughly a quarter of studies. While it was beyond the scope of this systematic review, it is likely that other sample characteristics (eg, ethnicity and socioeconomic status) are even less comprehensively reported, which makes it difficult to assess generalizability of neuro-oncology caregiver research. Fifth, there were two continents (Africa and South America) from which no publications were found. As many of the constructs which are important in family caregiving research are influenced by both healthcare systems and cultural factors, which can differ between countries let alone continents, future research should aim to include caregiver participants from Africa and South America as well.

In conclusion, neuro-oncology caregiving is a very active research field gaining more and more traction, but is still lagging behind research in other caregiver populations as well as neuro-oncology patient-focused research in terms of defining and establishing key constructs and consistent ways to assess these. Advancing the evidence-base further in a coordinated way, can aid the comparability of studies, limit research waste, and thus speed up progress which will directly benefit families living with primary brain tumors.

## Supplementary Material

npac058_suppl_Supplementary_Material_1

npac058_suppl_Supplementary_Material_2

npac058_suppl_Supplementary_Material_3
