# Supplementary material for: Family caregiver constructs and outcome measures in neuro-oncology: A systematic review
Source: Neurooncol Pract. 2022 Jul 20;9(6):465–74. doi: 10.1093/nop/npac058 (PMC9665052; doi:10.1093/nop/npac058)
Supplement: npac058_suppl_Supplementary_Material_1 [file npac058_suppl_supplementary_material_1.docx]

**Supplementary File 1**

PubMed search terms:

(("Outcome measures"[tw] OR "Outcome measure"[tw] OR "Outcome Assessment, Health Care"[Mesh:noexp] OR "Patient Reported Outcome Measures"[Mesh] OR "Surveys and Questionnaires"[Mesh] OR "Questionnaires"[tw] OR "Questionnaire"[tw] OR "Observer-reported outcome measure"[tw] OR "ObsRO"[tw] OR "Hospital Anxiety and Depression Scale"[tw] OR "HADS"[tw] OR "Center for Epidemiological Studies Depression Scale"[tw] OR "CES-D"[tw] OR "Caregiver Reaction Assessment"[tw] OR "CRA"[tw] OR "Mastery Scale"[tw] OR "Locke-Wallace Short Marital Adjustment Test for spousal relationships"[tw] OR "Caregiver QoL index-cancer"[tw] OR "CQOLC"[tw] OR "Caregiver Oncology QoL"[tw] OR "Caregiver Oncology QoL Questionnaire"[tw] OR "CarCOQol"[tw] OR "Short Form 36"[tw] OR "SF-36"[tw] OR "EuroQol"[tw] OR "European Quality of Life 5 Dimensions questionnaire"[tw] OR "EQ-5D"[tw] OR "Psychological Tests"[Mesh] OR "scale"[tw] OR "scales"[tw] OR "Interviews as Topic"[Mesh] OR "Interviews"[tw] OR "Interview"[tw] OR "Interview*"[tw] OR "self-report"[tw] OR "self-report"[tw] OR "self-reported"[tw] OR "self-report*"[tw]) AND ("outcome"[tw] OR "outcomes"[tw] OR "Psychological Distress"[Mesh] OR "Psychological Distress"[tw] OR "Psychologic Distress"[tw] OR "Stress, Psychological"[Mesh] OR "Psychological stress"[tw] OR "Psychologic stress"[tw] OR "Caregiver Burden"[Mesh] OR "burden"[tw] OR "burden*"[tw] OR "mental distress"[tw] OR "mental distress"[tw] OR "Quality of Life"[mesh] OR "Quality of Life"[tw] OR "QoL"[tw] OR "HRQoL"[tw] OR "life quality"[tw] OR "physical functioning"[tw] OR "physical function*"[tw] OR "Health Status"[mesh] OR "Health Status"[tw] OR "Physical Fitness"[Mesh] OR "Physical Fitness"[tw] OR "Physical Endurance"[Mesh] OR "Physical Endurance"[tw] OR "Recovery of Function"[Mesh] OR "Recovery of Function"[tw] OR "Caregiver mastery"[tw] OR "Patient-caregiver relationship"[tw] OR "family relationship"[tw] OR "Stress level"[tw] OR "Stress levels"[tw] OR "Coping"[tw] OR "Adaptation, Psychological"[Mesh] OR "General health"[tw] OR "Disease knowledge"[tw] OR "Social support"[tw] OR "Psychosocial support"[tw] OR "Social Support"[Mesh] OR "Uncertainty"[Mesh] OR "Uncertainty"[tw] OR "Health"[Mesh] OR "Self-wellbeing"[tw] OR "wellbeing"[tw] OR "well being"[tw] OR "Responsibilities"[tw] OR "Responsibility"[tw] OR "Obligation"[tw] OR "Obligations"[tw] OR "Social Responsibility"[Mesh] OR "Needs"[tw] OR "Productivity loss at work"[tw] OR "Productivity loss"[tw] OR "Absenteeism"[Mesh] OR "Absenteeism"[tw] OR "Employment status"[tw] OR "Employment"[Mesh] OR "Healthcare utilisation"[tw] OR "Health care utilisation"[tw] OR "Healthcare utilization"[tw] OR "Health care utilization"[tw] OR "Healthcare use"[tw] OR "Health care use"[tw] OR "Delivery of Health Care"[Mesh]) AND ("Caregivers"[Mesh] OR "Caregiver"[tw] OR "Caregivers"[tw] OR "Carers"[tw] OR "Carer"[tw] OR "Care Givers"[tw] OR "Care Giver"[tw] OR "Spouse Caregivers"[tw] OR "Spouse Caregiver"[tw] OR "Family Caregivers"[tw] OR "Family Caregiver"[tw] OR "Nuclear Family"[mesh] OR "Nuclear Family"[tw] OR "Parents"[tw] OR "Fathers"[tw] OR "Mothers"[tw] OR "Parent"[tw] OR "Mother"[tw] OR "Fathers"[tw] OR "Brothers"[tw] OR "Brother"[tw] OR "Sisters"[tw] OR "Sister"[tw] OR "Sons"[tw] OR "Son"[tw] OR "Daughters"[tw] OR "Daughter"[tw] OR "Siblings"[tw] OR "Sibling"[tw] OR "Spouses"[tw] OR "Spouse"[tw] OR "Husbands"[tw] OR "Husband"[tw] OR "Domestic Partners"[tw] OR "Domestic Partner"[tw] OR "Wives"[tw] OR "Wife"[tw] OR "next of kin"[tw] OR "Family"[Mesh] OR "Family"[tw] OR "Families"[tw**]** OR "Relatives"[tw] OR "Caretaker"[tw] OR "Caretaking"[tw] OR "Family support"[tw] OR "Proxy"[tw] OR "Companion"[tw] OR "Companion*"[tw] OR "informal care"[tw] OR "informal healthcare"[tw] OR "informal health care"[tw] OR "informal support"[tw]) AND ("Brain Neoplasms"[Mesh] OR "Glioma"[Mesh] OR "astrocytoma*"[tw] OR "astroglioma*"[tw] OR "brain cancer*"[tw] OR "brain carcinoma*"[tw] OR "brain malign*"[tw] OR "brain neoplasm*"[tw] OR "Brain Stem neoplasm"[tw] OR "Brain Stem neoplasms"[tw] OR "Brain Stem tumo*"[tw] OR "brain tumo*"[tw] OR "brainstem neoplasms"[tw] OR "brain-stem neoplasms"[tw] OR "brainstem neoplasm"[tw] OR "brain-stem neoplasm"[tw] OR "brainstem tumo*"[tw] OR "brain-stem tumo*"[tw] OR "cancer of brain"[tw] OR "cancer of the brain"[tw] OR "cancer of the central nervous system"[tw] OR "cancer of the cns"[tw] OR "central nervous system cancer*"[tw] OR "central nervous system malignan*"[tw] OR "central nervous system neoplasm*"[tw] OR "central nervous system tumo*"[tw] OR "Cerebellar cancer"[tw] OR "Cerebellar neoplasm*"[tw] OR "Cerebellar tumo*"[tw] OR "cerebral cancer*"[tw] OR "cerebral carcinoma*"[tw] OR "cerebral malignan*"[tw] OR "cerebral neoplasm*"[tw] OR "cerebral tumo*"[tw] OR "Choroid Plexus Papilloma*"[tw] OR "Choroid Plexus tumo*"[tw] OR "cns cancer*"[tw] OR "cns malignan*"[tw] OR "cns neoplasm*"[tw] OR "cns tumo*"[tw] OR "ependimom*"[tw] OR "ependymom*"[tw] OR "Ganglioglioma*"[tw] OR "glial cancer"[tw] OR "glial malign*"[tw] OR "glial neoplasm"[tw] OR "glial tumo*"[tw] OR "glioblastom*"[tw] OR "glioma*"[tw] OR "gliosarcoma*"[tw] OR "glyoma*"[tw] OR "Hypothalamic neoplasm*"[tw] OR "Hypothalamic tumo*"[tw] OR "Infratentorial cancer*"[tw] OR "Infratentorial Neoplasm*"[tw] OR "Infratentorial tumo*"[tw] OR "intracerebral cancer*"[tw] OR "intracerebral malignan*"[tw] OR "intracerebral neoplasm*"[tw] OR "intracerebral tumo*"[tw] OR "intracranial cancer*"[tw] OR "intra-cranial cancer*"[tw] OR "intracranial carcinoma*"[tw] OR "intracranial malignan*"[tw] OR "intracranial neoplasm*"[tw] OR "intracranial tumo*"[tw] OR " malignant brain*"[tw] OR "malignant glia*"[tw] OR "malignant primary brain*"[tw] OR "Medulloblastoma*"[tw] OR "Neurocytoma*"[tw] OR "neuroglioma*"[tw] OR "oligoastrocytoma*"[tw] OR "oligodendroblastoma*"[tw] OR "oligodendroglioma*"[tw] OR "oligoden-droglioma*"[tw] OR "oligo-dendroglioma*"[tw] OR "Pinealoma*"[tw] OR "Pinealomas"[tw] OR "Pituitary cancer*"[tw] OR "Pituitary neoplasm*"[tw] OR "Pituitary tumo*"[tw] OR "subependimom*"[tw] OR "subependymom*"[tw] OR "Supratentorial cancer*"[tw] OR "Supratentorial Neoplasm*"[tw] OR "Supratentorial tumo*"[tw] OR "tumor of brain"[tw] OR "tumor of central nervous system"[tw] OR "tumor of cns"[tw] OR "tumor of the brain"[tw] OR "tumor of the central nervous system"[tw] OR "tumor of the cns"[tw] OR "tumour of brain"[tw] OR "tumour of the brain"[tw] OR "tumour of the central nervous system"[tw] OR "tumour of the cns"[tw] OR "xanthoastrocytoma*"[tw] OR "xantoastrocytoma*"[tw] OR (("neoplasm*"[ti] OR "neo-plasm*"[ti] OR "tumor*"[ti] OR "tumour*"[ti] OR "cancer*"[ti] OR "malignan*"[ti]) AND ("glia*"[ti] OR "neuroglia*"[ti]))) NOT (("Infant"[mesh] OR "Child"[mesh] OR "Adolescent"[mesh] OR "Infan*"[ti] OR "Child*"[ti] OR "Adolescen*"[ti]) NOT ("Adult"[mesh] OR "Adult*"[ti] OR "elderly"[ti])) NOT (("Case Reports"[ptyp] OR "case report"[ti]) NOT ("Review"[ptyp] OR "review"[ti] OR "Clinical Study"[ptyp] OR "trial"[ti] OR "RCT"[ti] OR "case series"[ti])))
