# Supplementary material for: Family caregiver constructs and outcome measures in neuro-oncology: A systematic review
Source: Neurooncol Pract. 2022 Jul 20;9(6):465–74. doi: 10.1093/nop/npac058 (PMC9665052; doi:10.1093/nop/npac058)
Supplement: npac058_suppl_Supplementary_Material_3 [file npac058_suppl_supplementary_material_3.docx]

| 1. **Caregiver health** | | |
| --- | --- | --- |
| **CONSTRUCT: QUALITY OF LIFE** | | |
| **Instrument** | **Used >2 times** | **Used ≤2 times** |
| Caregiver QOL Index-Cancer scale (CQOLC) | X |  |
| Caregiver Oncology Quality of life questionnaire (CarGoQOL) |  | X |
| European Organisation for Research and Treatment of Cancer Quality of Life Questionnaire Core 30 (EORTC QLQ-C30) | X |  |
| EQ-5D |  | X |
| Fox Simple QOL |  | X |
| Functional Assessment of Cancer Therapy – General Population (FACT-GP) |  | X |
| Linear Analog Scale Assessment of Quality of Life (LASA) |  | X |
| Multidimensional Mood State Questionnaire (MDMQ) |  | X |
| National Institutes of Health Patient-Reported Outcomes Measurement Information System (NIH PROMIS) |  | X |
| Patient Generated Index |  | X |
| Schedule for the Evaluation of Individual QOL (SEIQoL) |  | X |
| Short-Form (SF-36 or SF-12) | X |  |
| Subjective Estimation of Quality of life (SQoL) questionnaire |  | X |
| Abbreviated World Health Organization Quality of Life (WHOQOL-BREF) |  | X |
| World Health Organization 5 (WHO-5) |  | X |
| **CONSTRUCT: WELLBEING OR GENERAL HEALTH** | | |
| Caregiver Quality of Life Index-Cancer Scale (CQOLC) |  | X |
| General Health Questionnaire (GHQ-12) |  | X |
| Health promoting lifestyle profile II (HPLP-II) |  | X |
| Karnofsky Performance Status (KPS) |  | X |
| Short-Form (SF-36) |  | X |
| Study-specific questionnaires |  | X |
| World Health Organization 5 (WHO-5) |  | X |
| **CONSTRUCT: DEPRESSION** | | |
| Beck Depression Inventory (BDI) |  | X |
| Center for Epidemiological Studies–Depression scale (CES-D) | X |  |
| Depression Anxiety and Stress Scale (DASS-42) |  | X |
| Emotion thermometers (E4) |  | X |
| ESAS (Psychological Distress Score (PSS) subscale) |  | X |
| General Health Questionnaire (GHQ-12) |  | X |
| Hospital Anxiety and Depression Scale (HADS) | X |  |
| Hamilton Depression Rating Scale (HAMD) |  | X |
| Patient Health Questionnaire (PHQ) |  | X |
| Study-specific questionnaires |  | X |
| **CONSTRUCT: ANXIETY** | | |
| Death Anxiety Scale (DAS) |  | X |
| Depression Anxiety and Stress Scale (DASS-42) |  | X |
| Emotion Thermometers (E4) |  | X |
| ESAS (Psychological Distress Score (PSS) subscale) |  | X |
| General Anxiety Disorder (GAD), 2 or 7 item |  | X |
| General Health Questionnaire (GHQ-12) |  | X |
| Hospital Anxiety and Depression Scale (HADS) | X |  |
| Hamilton Anxiety Rating Scale (HAMA) |  | X |
| Profile of Mood States (POMS) |  | X |
| Self-rating anxiety scale (SAS) |  | X |
| Spielburger State-Trait Anxiety Inventory (STAI-S / STAI-T) |  | X |
| Visual Analogue Scale (VAS) |  | X |
| **CONSTRUCT: DISTRESS** | | |
| Brain Injury Community Rehabilitation Outcome Scale (BICRO-39), Psychological and Behavioural adjustment subscale (adapted) |  | X |
| Caregiver Quality of Life Index-Cancer Scale (CQOLC) |  | X |
| Caregiver Risk Screen |  | X |
| Depression Anxiety and Stress Scale (DASS-42) |  | X |
| Distress thermometer | X |  |
| Emotion thermometers (E4) |  | X |
| Family Appraisal of Caregiving Questionnaire for Palliative Care (FACQ) |  | X |
| General Health Questionnaire (GHQ) | X |  |
| Hospital Anxiety and Depression Scale (HADS) |  | X |
| Hornheider Questionnaire (Adapted) |  | X |
| Neuropsychiatric Inventory Questionnaire (NPI-Q) |  | X |
| Patient Health Questionnaire (PHQ) 2 or 9 item |  | X |
| Perceived Stress Scale (PSS-10) | X |  |
| Profile of Mood States |  | X |
| Short-Form-12 Mental Component Summary (SF-12v1) |  | X |
| Study-specific questionnaires |  | X |
| Abbreviated World Health Organization Quality of Life (WHOQOL-BREF) (psychological domain) |  | X |
| **CONSTRUCT: MOOD OR SPECIFIC EMOTIONS** | | |
| Emotion thermometer |  | X |
| (Adapted) Fear of Cancer Recurrence 7 item (FCR-7) |  | X |
| Profile of Mood States (POMS) |  | X |
| Visual Analogue Scale (VAS) |  | X |
| **CONSTRUCT: PHYSICAL HEALTH** | | |
| Brief Pain Inventory |  | X |
| Caregiver Quality of Life Index-Cancer Scale (CQOLC) |  | X |
| Caregiver Reaction Assessment (CRA) |  | X |
| Edmonton Symptom Assessment System (ESAS) |  | X |
| Short-form (SF-36 or SF-12 or subscale) | X |  |
| Study-specific questionnaires |  | X |
| **CONSTRUCT: SLEEP OR FATIGUE** | | |
| Attentional Function Index |  | X |
| Brief Fatigue Inventory |  | X |
| Checklist Individual Strength (CIS) |  | X |
| General Sleep Disturbance Scale (GSDS) |  | X |
| Insomnia Severity Index |  | X |
| Lee Fatigue Scale |  | X |
| Pittsburgh Sleep Quality Index (PSQI) | X |  |
| 1. **Caregiver needs** | | |
| **CONSTRUCT: (UNMET) SUPPORT NEEDS** | | |
| Access to Services Needs Scale |  | X |
| Brief Social Support Questionnaire (BSSQ) |  | X |
| Brain Tumour Specific Supportive Care Needs Scale (BrTSCNS) |  | X |
| (Adapted) Canadian Problem Checklist (A-CPC) |  | X |
| Caregiver Needs Assessment |  | X |
| Caregiver Needs Screen |  | X |
| Caregivers Perspective Questionnaire (CPQ), based on PPQ (adapted) |  | X |
| Carer Support Needs Assessment Tool (CSNAT) |  | X |
| Distress Thermometer |  | X |
| Partner and Caregiver Supportive Care Needs Scale (SCNS-P&C44) |  | X |
| Social Support Questionnaire (F-SozU) |  | X |
| Study-specific questionnaires |  | X |
| **CONSTRUCT: (UNMET) INFORMATION NEEDS** | | |
| Access to Services Needs Scale |  | X |
| Mode/Format Preferences to Receiving Information |  | X |
| Partner and Caregiver Supportive Care Needs Scale (SCNS-P&C44) |  | X |
| Patient Information Needs Questionnaire |  | X |
| Prognostic and Treatment Perception Questionnaire |  | X |
| Study-specific questionnaires |  | X |
| **CONSTRUCT: OTHER SUPPORT** | | |
| Iceland-Family Perceived Support Questionnaire |  | X |
| Interpersonal Support Evaluation List (ISEL) |  | X |
| 1. **Caregiver tasks** | | |
| **CONSTRUCT: CAREGIVER BURDEN** | | |
| Burden Scale for Family Caregivers (BSFC) |  | X |
| Caregiver Burden Scale (CBS) |  | X |
| Caregiver Quality of Life Index-Cancer Scale (CQOLC) |  | X |
| Caregiver Reaction Assessment (CRA) | X |  |
| Caregiver Strain Index (adapted) |  | X |
| Family Appraisal of Caregiving Questionnaire for Palliative Care (FACQ) |  | X |
| Study-specific questionnaires |  | X |
| Zarit Burden Interview (ZBI) | X |  |
| **CONSTRUCT: IMPACT OF CARING** | | |
| Caregiver Strain Index |  | X |
| Family Appraisal of Caregiving Questionnaire for palliative care (FACQ-PC) |  | X |
| Study-specific questionnaires |  | X |
| 1. **Caregiver beliefs and attitudes** | | |
| **CONSTRUCT: COPING** | | |
| Brief Religious Coping Scale (BriefCOPE) |  | X |
| Brief Coping Orientation to Problems Experienced Scale (BriefCope) |  | X |
| Caregiver Coping Questionnaire (CCQ) |  | X |
| Measure of Coping Status-A (MOCS-A) |  | X |
| Study-specific questionnaires |  | X |
| SWB Linear Analog Self-Assessment |  | X |
| **CONSTRUCT: CAREGIVER MASTERY** | | |
| Caregiver Mastery Scale | X |  |
| Study-specific questionnaires |  | X |
| **CONSTRUCT: PREPAREDNESS FOR CARING** | | |
| Preparedness for Caregiving Scale | X |  |
| Study-specific questionnaires |  | X |
| **CONSTRUCT: SELF-EFFICACY** | | |
| Caregiver Competence Scale |  | X |
| Revised Caregiver Self-Efficacy Scale (CSES-R) |  | X |
| Short General Self-Efficacy Index |  | X |
| **CONSTRUCT: RESILIENCE** | | |
| Cognitive and Affective Mindfulness Scale Revised |  | X |
| Family Hardiness Index (FHI) |  | X |
| Short-Form 12v2 |  | X |
| **CONSTRUCT: POSITIVE ASPECTS OF CARING** | | |
| Family Appraisal of Caregiving Questionnaire for palliative care (FACQ-PC) |  | X |
| **CONSTRUCT: SPIRITUALITY** | | |
| Functional Assessment of Chronic Illness Therapy-Spiritual Expanded Version (FACIT-SP-Ex) |  | X |
| Study-specific questionnaires |  | X |
| 1. **Environment** | | |
| **CONSTRUCT: SPECIFIC EXPERIENCES** | | |
| ADL scale |  | X |
| Caregiver Reaction Assessment (CRA) |  | X |
| Cognitive Affective mindfulness scale-revised |  | X |
| Family Decision Making Self-efficacy scale (FDMSES) |  | X |
| Impact of Event Scale-Revised (IES-R) |  | X |
| Isolation questionnaire |  | X |
| Prognosis and Treatment Perceptions Questionnaire (PTPQ) |  | X |
| Revised Bakas Caregiving Outcomes Scale |  | X |
| Study-specific questionnaires |  | X |
| **CONSTRUCT: RELATIONSHIP OR FAMILY FUNCTIONING** | | |
| Caregiver QOL Index-Cancer (CQOLC) |  | X |
| Family APGAR Index |  | X |
| Family Appraisal of Caregiving Questionnaire for Palliative Care (FACQ) |  | X |
| Family Environment Scale (FES) |  | X |
| Family Function Style Scale (FFSS) |  | X |
| Iceland-Expressive Family Functioning Questionnaire |  | X |
| Intimate Bond Measure (IBM) |  | X |
| Lock-Wallace short marital adjustment scale |  | X |
| Social Support Scale |  | X |
| Study-specific questionnaires |  | X |
| **CONSTRUCT: PAID WORK** | | |
| Caregiver Strain Index |  | X |
| Study-specific |  | X |
| Work Limitations Questionnaire |  | X |
| **CONSTRUCT: FINANCIAL ISSUES** | | |
| Caregiver Quality of Life Index-Cancer Scale |  | X |
| Caregiver Reaction Assessment (CRA) |  | X |
| Caregiver Strain Index |  | X |
| Economic Hardship Questionnaire |  | X |
| Study-specific |  | X |
| Trimbos iMTA Questionnaire for Costs associated with psychiatric illness (TIC-P) |  | X |
| 1. **Other constructs** | | |
| **CONSTRUCT: PREFERENCES** | | |
| Study-specific questionnaires |  | X |
| **CONSTRUCT: SATISFACTION OR EVALUATION** | | |
| Canadian Health Care Evaluation project Questionnaire |  | X |
| Study-specific questionnaires |  | X |
| Toolkit After Death Bereaved Family member Interview |  | X |
| **CONSTRUCT: CAREGIVER PERSONALITY TRAITS** | | |
| Goldberg Adjective Scale (neuroticism) |  | X |
